# Supplementary material for: Predicting unplanned readmissions in the intensive care unit: a multimodality evaluation
Source: Sci Rep. 2023 Sep 18;13:15426. doi: 10.1038/s41598-023-42372-y (PMC10507073; doi:10.1038/s41598-023-42372-y)
Supplement: Supplementary file 1 — Supplementary Information. [file 41598_2023_42372_MOESM1_ESM.docx]

A TRAINING Details

All models (except the RF model) were trained using a singleNVIDIA A-100 GPU. To avoid over-fitting, all models used a stop loss of 7 instead of an epoch limitation.We consider a model to have improved if the AUPRC score for the validation set has risen between two successive evaluations, conducted every 200 steps. If the answer is positive we save the model, otherwise we reduce the learning rates. If the stop loss has been exceeded, we stop the training and return the last saved model. We then use the saved model to evaluate it on the test set. We used a batch size of 64. The NLP models use a learning rate of 2 · 10^−5^, and a reduction rate of 0.9, the rest use 10^−3^ and 0.97 respectively. For each model we perform this training loop for each of the five folds.
